# Topological Navigation for Autonomous Underwater Vehicles in Confined Semi-Structured Environments

**DOI:** 10.3390/s23052371

**Published:** 2023-02-21

**Authors:** Claudio Rossi, Adrian Caro Zapata, Zorana Milosevic, Ramon Suarez, Sergio Dominguez

**Affiliations:** Centre for Automation and Robotics UPM-CSIC, Universidad Politécnica de Madrid, 28006 Madrid, Spain

**Keywords:** underwater robots, 3D navigation, topological navigation, labeled node matching

## Abstract

In this work, we present the design, implementation, and simulation of a topology-based navigation system for the UX-series robots, a spherical underwater vehicle designed to explore and map flooded underground mines. The objective of the robot is to navigate autonomously in the 3D network of tunnels of a semi-structured but unknown environment in order to gather geoscientific data. We start from the assumption that a topological map has been generated by a low-level perception and SLAM module in the form of a labeled graph. However, the map is subject to uncertainties and reconstruction errors that the navigation system must address. First, a distance metric is defined to compute node-matching operations. This metric is then used to enable the robot to find its position on the map and navigate it. To assess the effectiveness of the proposed approach, extensive simulations have been carried out with different randomly generated topologies and various noise rates.

## 1. Introduction

The autonomous navigation and mapping of flooded underground mines is a very complex task. These are made up of a complex 3D network of tunnels and shafts that constitute an extremely hazardous environment for human divers, which in any case could only reach a relatively limited depth range, much smaller than that of a standard mine [1]. Therefore, the use of underwater vehicles (UVs) appears as a natural alternative to overcome the drawbacks of direct human exploration. Due to the depth and extension of the network, remote-operated underwater vehicle (ROV) technology using tethered vehicles is also ruled out. The dimensions and complexity of the mines and, more importantly, the risk of entanglement in protruding elements of the old mine infrastructure will most certainly put the mapping mission in danger.

Other methods, such as the deployment of ad hoc communication relay nodes, have been proposed, e.g., breadcrumb networks [2]. However, they could not be considered suitable for flooded mine exploration for a number of reasons. First, the topology of the mine tunnels and shafts is not known a priori, making the deployment of the beacon strategy too complex. Furthermore, the precarious structural state of the mines discourages any contact with their walls due to the risk of collapse. Finally, the deployed nodes could represent obstacles for the movement of the submarine itself and an unacceptable alteration of the natural environment if left abandoned (many sites are considered archaeological sites and therefore protected as historical heritage).

**Figure 1:**
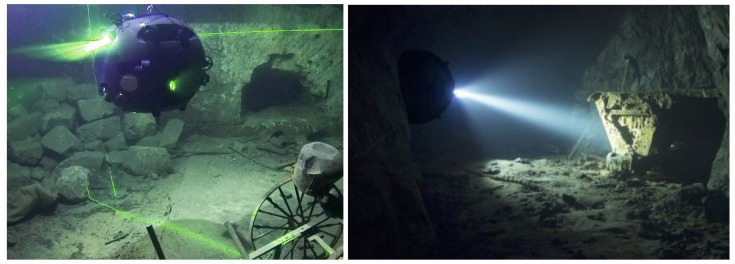
The UX-1 robot and examples of visual landmarks (“OOIs - Object of Interests”) that can be used for robot self-localisation.

Given all these limitations, the use of autonomous robots appears to be the only suitable choice. The UNEXMIN (H2020, Grant agreement No 690008. www.unexmin.eu, accessed 20 February 2023) and UNEXUP (EIT Raw Materials, Grant agreement No. 19160. www.unexup.eu, accessed 20 February 2023) projects have the motivation to overcome these challenges. The main objective of the UNEXMIN project was to develop a team of autonomous underwater vehicles (AUVs), named UX-class underwater explorers (see Figure 1, left), specifically designed to explore and map underground flooded mines. The UNEXUP project focuses on bringing the technology to the market while improving the robot’s capabilities. The UX-series robots developed in these two projects are equipped with navigation instruments that allow them to build a mine map as they pass through tunnels and collect geological information using their scientific instruments. (More details on the hardware architecture of the UX-1 can be found in [3]).

A map of the mine is generated using the topological information previously collected and the information provided by the low-level sensor fusion and Simultaneous Localization And Mapping (SLAM) software module, implementing techniques such as hybrid visual SLAM [4], direct or semi-direct SLAM [5,6], or others (e.g., [7,8,9]). This map is composed of processed geometry information (Octomap [10]) and a topology graph, where the former is used for guidance and reactive planning (keeping the center of the tunnel, avoidance of obstacles, see [11]). The topological graph consists of a set of labeled nodes (representing tunnel crossings) and labeled arcs (representing mine tunnels). This is used to plan the high-level exploration strategy, i.e., the sequence of nodes to visit.

The main objective of this work is to implement the autonomous navigation system of the UX-series robots under the following assumptions:

- Odometry measurements cannot be used because they rapidly become unreliable due to drift;
- Prior topological information is available, either from maps or previous dives;
- Both arcs and nodes of the graph have associated useful information that can be used to localize the robots, including both geometrical information and visual landmarks;
- Such information can be noisy and prone to error.

From these assumptions, the following research goals are addressed:

- To develop a robust on-board node-matching strategy in order to compute the matching between the run-time perceived information and the pre-defined map;
- To use such information for localization and dead-reckoning;
- To test the proposed strategies under different noise levels.

This paper is organized as follows: Section 2 reviews related work in the field of topology-based navigation, with a special emphasis on three-dimensional navigation and underwater robots. Section 3 describes the encoding of the topology information in the navigation graph, and Section 4 describes the *node matching* metric devised to address uncertainties and possible perception errors of the information associated with the graph nodes. Section 5 reports on the tests performed to assess the effectiveness of the algorithms developed in randomly generated mine topologies. Finally, the conclusions drawn from the work carried out and additional notes on future steps toward the development of the system are presented in Section 6.

## 2. Related Work

Unmanned UVs have many potential applications in a variety of fields, such as ocean mining exploration [12], autonomous seafloor mapping [13,14], data collection [15,16,17], maritime security [18], marine archaeology [19,20], and search and rescue [21].

The literature related to underwater robotics is rather vast, centering on diverse aspects such as mechanical design [22], modeling and control [23], navigation [24], perception [25], multi-robot coordination [26], and manipulation [27]. In this paper, we focus on navigation. Classical approaches to this problem include inertial navigation, geophysical navigation, terrain-assisted navigation, geomagnetism-assisted navigation, and acoustic navigation technology [28]. All such strategies have been developed for navigation in open waters. This work differs from most of the work in the state of the art in that it focuses on highly confined spaces.

### 2.1. Autonomous Navigation in Confined Underwater Environments

Although underwater navigation is a relatively consolidated field of research, most of the work in this field addresses open-water environments [29] or, alternatively, partially confined environments such as submarine canyons or water masses under layers of superficial ice [30,31], or environments with dense obstacles [32].

Various comprehensive surveys can be found on the autonomous exploration of confined underground spaces on land, such as [33]; however, the body of literature that addresses the exploration of confined underwater spaces, such as flooded caves and tunnels, narrows dramatically. Work on marine archaeology and geological prospecting can be found in [34]. In [35], the problem of navigation in confined underwater spaces for debris detection is addressed using occupancy grid maps.

Underwater path planning has often been addressed in the literature as a 2D problem, adapting work from terrestrial path planning [29]. In [36] a framework is proposed for the autonomous exploration of confined indoor environments that share several key characteristics with the exploration of underwater tunnels. A semantic road map that represents the topology is built incrementally. Certain related approaches have also been proposed, considering equivalence classes of maps and incorporating specifics of underwater navigation, such as current forces [37]. The environments targeted here require 3D exploration to capture tunnel and shaft structures, and solutions for the flat 2D problem are not easily expandable to 3D.

Due to the extension of the environment and its complexity, grid-based and odometry-based navigation methods have been ruled out for the application at hand. Topology-based navigation has the advantage of requiring an accurate determination of the robot’s pose, and odometry is more efficient in terms of space and computational resources needed and provides a convenient representation for symbolic planners/problem solvers. On the other hand, topological maps may be sensitive to noise and uncertainties as a result of low-level perception software layers.

In a similar context, Akbari et al. [38] propose a “frontier exploration” approach, where the frontier is the boundary between the explored and unexplored region. In their approach, the knowledge of the environment is structured through a semantic network composed of nodes, which are regions, objects or other concepts, and links which express the relations between them. Navigation through the network is performed by an ad hoc heuristic algorithm that does not take into account possible uncertainties or errors.

### 2.2. Labeled Subgraph Matching

In this work, we assume that a topology graph has previously been built. To this aim, a variety of methods have been proposed in the literature (see, e.g., [39]).

Hence, this work is framed in the labeled (sub)graph-matching field, also known as the subgraph isomorphism problem. This is a well-known problem in the field of pattern matching, and several algorithms have been proposed, although mostly for perfect subgraph matching in unlabeled graphs. This problem is known to be NP-complete. The matching problem can also be inexact, allowing for missing nodes and/or edges or different node and/or edge labels [40,41]. The literature on the topic of subgraph isomorphism is fairly vast, and the first references to this problem date back to 1967 [42]. However, the work presented here differs from other work in this field in three ways.

First, most of the labeled graphs, by “label”, refer to a single field (e.g., a symbol or a number, see, e.g., [43]), while in our case labels are fairly complex, containing much more information that is used for evaluating the goodness of a matching.

Second, our algorithm follows the path navigation logic and is, therefore, incremental. Therefore, the problem is reduced to a single-node matching problem with accumulated uncertainty, rather than a complete graph matching (also known as “query graph” in the literature).

Finally, we consider that the information associated with the nodes (the label) can be inexact, a fact not considered in labeled graph matching algorithms.

To the best of our knowledge, due to these three specific features, no algorithm in the state of the art is applicable to our case.

## 3. Graph Topology

**Figure 2:**
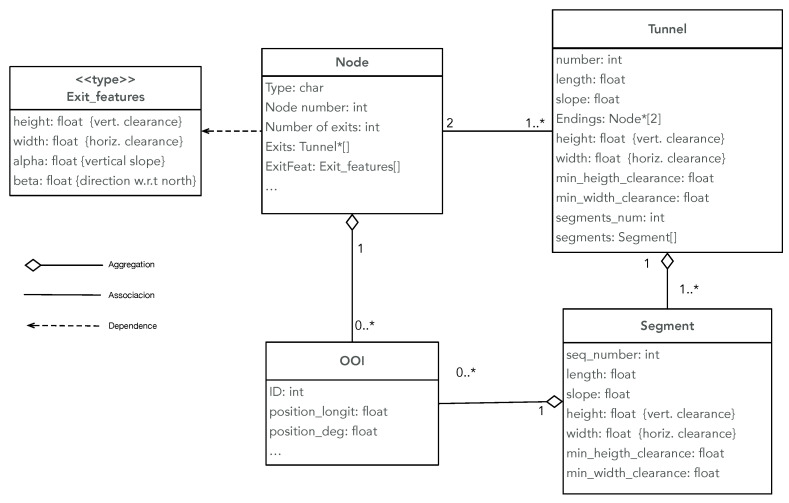
Diagram of the entities in the map graph. The main classes and their relationship are displayed using the UML language. Only the attributes used in this work are shown.

Figure 2 shows the UML diagram of the information related to the nodes and tunnels. At this stage of our work, for navigation purposes, only the information of the nodes is taken into account.

Nodes are labeled with the following information:

- Type: “E” (entrance), “X” (dead end), “U” (uncharted), “C” (crossing);
- Sequence number;
- Number of exits;
- Geometric position;
- List of incident tunnels;
- List of features associated to each exit (estimated height, width, and angles α (with regard to north) and β (with regard to the horizontal plane).

Tunnels are composed of segments and are labeled with the following information:

- Sequence number;
- Estimated length;
- Average slope;
- List of incident nodes;
- Average horizontal and vertical clearance;
- Minimum horizontal and vertical clearance;
- Number and list of segments.

Straight tunnels are composed of only one segment. Multiple segments are used to define different sections of long tunnels that differ by some geometrical property or by a different direction.

Finally, both nodes and tunnels can be associated with objects of interest (OOI). OOI are objects recognized and registered during the mission, such as signs and objects left behind when the mine was abandoned (trolleys, tools, etc., see Figure 1).

**Figure 3:**
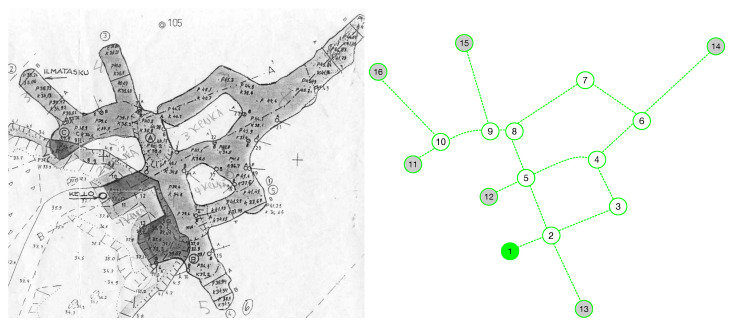
(**Left**): handwritten map of Kaatiala mine. (**Right**): topology graph of the mine. Gray note correspond to dead ends, and the green node is the mine entrance node.

Figure 3 shows an example of a real mine (Kaatiala mine, Finland). On the left, a handwritten map produced by human scuba divers is shown. On the right, the corresponding topology graph (extracted by hand) is shown. Label “C” means crossing (“normal” node), “E” means the entrance of the mine, label “X” means “dead end”, and label “U” means uncharted. Note that sharp angles are treated as nodes.

All the information associated with the nodes and tunnels comes from low-level perception and sensor fusion software and is therefore approximated and prone to errors. For example, the tunnel length is determined by odometry, and the number of tunnels departing from a node shall be computed by the on-board software. On occasion, hand-made maps are available.

A navigation plan consists of a sequence of nodes to be visited until the robot reaches either the given point for gathering further information (using scientific devices such as a multispectral camera, water sampler, gamma-ray spectrometer, etc.) or the end of the explored area (“U” node), from which to start mapping an uncharted part and enlarge the map. The robot uses the nodes’ information as a checkpoint to localize itself during navigation.

## 4. Proposed Node-Distance Metric

As mentioned above, the information coming from the SLAM module can be noisy, and the information associated with the graph nodes and arcs can be imprecise. This applies both to the topological map and to the real-time sensory information provided to the robot. Therefore, finding a perfect match between the current node and the map nodes is rarely possible.

For the best node-matching estimation, we have developed a distance metric that considers possible errors in the data. Such errors are related to the number of arcs incident to each node (number of tunnels in the crossing), the dimension of the tunnels and their orientation, as well as entrance information (height and width).

When the robot reaches a crossing, the SLAM module detects the number of tunnels in the crossing and their main features.

- *H*: height of the tunnel;
- *W*: width of the tunnel;
- α: direction of the tunnel with regard to the north;
- β: inclination of the tunnel with regard to the horizontal plane.

Let *s* be the perceived node, and *m* be a node on the map. Let $N^{s}$ be the number of tunnels leaving (incident arcs) from node *s* and $N^{\left(m\right)}$ be the number of tunnels leaving map node *m*.

If both nodes have the same number of exit tunnels (incident arcs) $N^{\left(s\right)}=N^{\left(m\right)}=N$, then for each tunnel *i* departing from a node, from left to right, we take into account the following indices (difference between features):

$$dH_{i}=\frac{H_{i}^{s}-H_{i}^{m}}{max(H_{i}^{s},H_{i}^{m})}$$

$$dW_{i}=\frac{W_{i}^{s}-W_{i}^{m}}{max(W_{i}^{s},W_{i}^{m})}$$

$$d\alpha_{i}=\frac{\alpha_{i}^{s}-\alpha_{i}^{m}}{max(\alpha_{i}^{s},\alpha_{i}^{m})}$$

$$d\beta_{i}=\frac{\beta_{i}^{s}-\beta_{i}^{m}}{max(\beta_{i}^{s},\beta_{i}^{m})}$$

and define the matching between tunnel information:

$$P_{i}=\left(1-dH_{i}\right)\left(1-dW_{i}\right)\left(1-d\alpha_{i}\right)\left(1-d\beta_{i}\right)$$

(note that all these indices take values in the range [0,1], where 0 indicates a bad match and 1 a perfect match). Then,

$$E_{s,m}=\frac{\sum_{i=1}^{N}P_{i}}{N}$$

is the global similarity index between node *m* and node *s*.

If the two nodes do not have the same number of incident arcs, a penalty term, empirically set, $E_{s,m}=0.2$ is used.

The robot uses the calculated indices *E* to confirm its location during navigation. Additionally, this information is used to update the accumulated confidence index $I^{\left(t\right)}$:

$$I^{\left(t\right)}=I^{\left(t-1\right)}\cdot E_{s,m}\;,$$

where $I^{\left(0\right)}=1$. Therefore, the robot’s confidence that it is at the node *m* of the map depends on the accumulated certainty. In other words, its confidence depends on the path it has followed. The confidence index *I* can be reset in determined situations when the robot’s self-localization is considered to be very reliable (see below).

### Navigation Procedure

**Figure 4:**
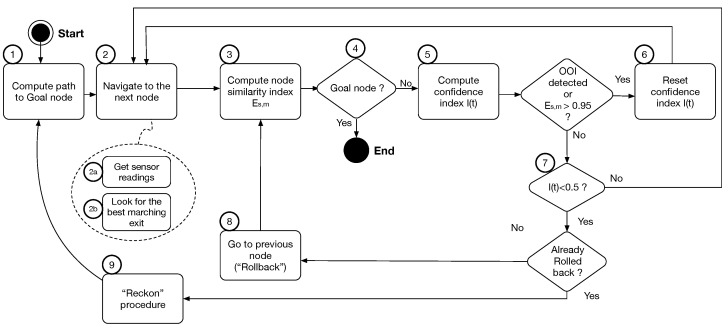
Navigation procedure flow chart (see text below for a detailed description). Step 2 is composed of two sub-steps.

The navigation procedure is as follows (see Figure 4):

- When reaching node *s*, the index $E_{s,m}$ is calculated for the *perceived* node (*s*) and the *expected* node *m* in the map graph (Step 3).
- If the goal node is detected, the procedure ends (Step 4).
- If not, the confidence level I(t) is calculated (Step 5).
- If the node matching estimation produces a very high number (threshold set to 0.95), then $I^{\left(t\right)}$ is reset to 1, since an almost perfect match indicates a high reliability of the sensory data. Additionally, when the robot detects and recognizes an object of interest (OOI), the index $I^{\left(t\right)}$ is reset to 1 since this indicates extremely high confidence in the robot’s position (Step 6).
- When $I^{\left(t\right)}$ drops below a certain threshold (set to 0.5 in the experiments), the sensory information is considered unreliable; in other words, the robot cannot have confidence in its perception of the environment and is considered to be lost (Step 7).
- In this case, the following reckon procedure is run. First, the robot returns to the previous node and repeats the tunnel exit selection (“*Rollback*” step) (Step 8). If it takes the correct exit (that is, the destination node matches the expected one) and the navigation continues normally. If the node reached again has a bad matching index, it searches the whole map trying to recognize the node where it is (“*Reckon*” step). The index *E* is computed for all the nodes on the map (with regard to the node the robot currently “sees”), and the robot assumes to be at the node with a higher index (Step 9).

Of course, this may not be the node where the robot is in fact located. In this case, it is likely that at the index m,m+1,... of the following nodes *E*, and hence $I^{\left(t\right)}$, the index would drop dramatically since the expected nodes have nothing to do with the perceived ones. The procedure would then be repeated.

## 5. Experiments

To assess the effectiveness of the proposed approach, a large number of simulations of randomly generated graphs have been performed.

For all the tests, the nodes were generated using the following parameters:

- Number of tunnels departing from each node: 2–4;
- Tunnel entrance height: 1.0–1.5 m;
- Tunnel entrance width: 1.5–2.0 m;
- Tunnel vertical orientation (β): 0–360 degrees;
- Tunnel horizontal orientation (α): 0–360 degrees.

### 5.1. Lost Robot Experiments

To test the robot’s ability to find itself after entering the lost mode and the effectiveness of the node matching algorithm, we run a series of tests deploying the robot at a random unknown node and running the “*reckon*” step described above.

The perception of the characteristics of the nodes is a critical factor. To take into account perception errors, a random error is applied to the sensors’ readings. Varying values of the random error have been used: ±5%,±10%,±25%, and ±50%. This test algorithm has been run for different network sizes (20, 50, 100, and 150 nodes), running 500 tests for each randomly generated graph. The correctness of the node matching is confirmed by navigating to the next node and repeating the node matching process to verify whether this node matches the expected destination. In the case of an unsuccessful confirmation, the above steps are repeated from the (incorrect) destination node.

Table 1 reports the results of the node matching algorithm. The left column of each graph size reports the success rate of the first attempt. The success rate is around 90% or higher in most cases. Only for a noise level of 50% this decreases significantly. It is worth noting, however, that such a noise rate is extremely high: for instance, a tunnel width of 1 m can be perceived as 0.5 m or 1.5 m.

The right column of each graph size reports the success rate on the second attempt, showing how the success rate is improved by repeating the procedure. In principle, this can be repeated until the robot eventually finds its correct position. In the navigation tests performed (see the next section and Table 2), such repetitions were limited to five, until the robot was declared lost.

**Table 1:** Lost robot test: success rate of the node-matching procedure.

Noise	Graph Size (Nodes)									
Rate	20		50		100		150		200	
5%	93.7%	95.0%	95.5%	96.1%	95.3%	99.1%	94.1%	95.5%	94.1%	96.2%
10%	88.2%	91.2%	95.0%	97.1%	96.2%	98.5%	86.8%	89.7%	91.0%	91.9%
25%	88.8%	92.5%	90.6%	96.9%	89.7%	93.8%	87.7%	96.3%	87.8%	95.9%
50%	71.9%	86.1%	69.8%	86.5%	61.9%	84.5%	55.2%	82.2%	51.5%	77.4%

### 5.2. Navigation Tests

For navigation tests, a total of 400 graphs with sizes 25, 50, 100, and 150 nodes were generated using the parameters described above. For each graph, a path from the start to the goal node, randomly chosen, was generated using the well-known Dijkstra algorithm. The generated graphs are always connected, so there is always a path.

Navigation tests have been carried out to assess the effect of noise on sensor readings. Random noise of ±5%,±10%,±15%,±20%,±25% were added to all the parameters described above. This affects both tunnel selection during navigation (the robot may take the wrong exit due to incorrect or imprecise sensor readings) and the node-matching procedure.

The first set of runs aimed to test navigation without OOI detection. Table 2 summarizes the results of these tests. As can be seen, the success rate is almost always 100% and decreases for high noise rates. Evidently, the probability of being lost increases for long path lengths and high noise rates. This can also be seen in the increasing values of time when the two steps of the reckoning procedure are activated (“Reckon” column).

In most cases, the robot was able to navigate successfully from the starting node to the end node, except for high noise levels, which led to high uncertainties. Table 2 also summarizes the success rate (percentage of times the algorithm found the correct path) and the final average value of the uncertainty index $I^{\left(t\right)}$ at the end of the run.

**Table 2:** Results of the path navigation and reckoning procedure.

Number	Noise	Path	Success	Final Confidence		Rollbacks		Reckon	
of Nodes	Level	Length	Rate	Avg.	Std. Dev.	Avg.	Std. Dev.	Avg.	Std. Dev.
25	0.05	5.83	100%	0.87	0.08	0.05	0.22	0.03	0.18
25	0.10	5.59	100%	0.51	0.21	0.71	1.44	0.13	0.34
25	0.15	5.59	100%	0.47	0.17	0.94	0.74	0.57	0.67
25	0.20	5.67	100%	0.47	0.16	1.53	0.87	1.10	0.75
25	0.25	5.30	98%	0.40	0.15	2.00	1.11	1.50	1.23
50	0.05	9.60	100%	0.87	0.08	0.03	0.17	0.00	0.00
50	0.10	9.62	100%	0.52	0.19	1.40	1.26	0.81	0.64
50	0.15	9.85	100%	0.50	0.18	1.95	0.97	1.63	0.96
50	0.20	9.96	100%	0.50	0.16	3.00	1.32	2.57	1.28
50	0.25	9.00	81%	0.40	0.22	2.80	1.86	3.60	1.72
100	0.05	18.52	100%	0.84	0.11	0.08	0.35	0.02	0.14
100	0.10	17.31	100%	0.48	0.18	6.52	5.63	1.98	1.19
100	0.15	12.81	79%	0.36	0.24	2.90	1.96	3.82	1.71
100	0.20	15.74	70%	0.24	0.25	1.75	2.07	4.49	1.89
100	0.25	16.60	36%	0.10	0.21	1.10	1.98	4.40	1.27
150	0.05	26.68	100%	0.85	0.10	0.11	0.49	0.04	0.19
150	0.10	21.14	91%	0.43	0.24	9.08	6.65	4.25	2.80
150	0.15	28.18	61%	0.26	0.28	1.84	2.06	4.63	2.02
150	0.20	23.41	45%	0.11	0.21	1.09	2.07	4.50	1.08
150	0.25	21.50	27%	0.10	0.15	0.80	1.49	4.70	0.96

### 5.3. Use of OOI to Aid Navigation

This set of tests aims to evaluate the effect of OOI on aiding localization. OOI were randomly generated and added to the maps. Their actual recognition was also affected by different noise levels.

Table 3 summarizes the final value of the index *I* at the end of the runs for various combinations of the P(OOI) and P(detection) parameters. As expected, improved OOI detection, either due to a better detection probability or because they are present in a higher number, improves the reliability of the robot’s self-localization, and, therefore, the whole navigation process. This is quantitatively reported in the value of the *I* index: higher values indicate greater confidence in the localization (let us recall that the correct detection of an OOI implies resetting the confidence index *I*).

Table 4 provides details of the results for a given combination of parameters (probability of the presence of OOI, P (OOI) = 0.2 and probability of detection of OOI, P (detection) = 0.9), reporting the success rate, final confidence, as well as the number of *Rollbacks* and *Reckon* operations.

**Table 3:** Impact of OOI detection on navigation and localization (summary of final index values *I*).

	Probability of OOI									
Prob. of	0.05		0.1		0.2		0.4		0.5	
Detection	Avg.	Std.Dev.	Avg.	Std.Dev.	Avg.	Std.Dev.	Avg.	Std.Dev.	Avg.	Std.Dev.
0.25	0.81	0.31	0.83	0.28	0.85	0.27	0.88	0.21	0.88	0.22
0.5	0.83	0.29	0.83	0.27	0.86	0.24	0.88	0.27	0.89	0.26
0.75	0.83	0.28	0.85	0.26	0.89	0.21	0.91	0.21	0.92	0.21

**Table 4:** Impact of OOI detection on navigation, for P (OOI) = 0.2 (approximately 20% of nodes that contain recognizable features), and P(detection) = 0.9.

Number	Noise	Path	Success	Final Confidence		Rollbacks		Reckon		OOI Detections	
of Nodes	Level	Length	Rate	Avg.	Std.Dev.	Avg.	Std.Dev.	Avg.	Std.Dev.	Avg.	Std.Dev.
25	0.05	5.61	100%	0.89	0.08	0.00	0.00	0.00	0.00	0.74	0.97
25	0.1	5.65	100%	0.66	0.21	0.22	0.86	0.05	0.28	1.00	1.03
25	0.15	5.67	100%	0.60	0.26	0.61	0.60	0.26	0.44	0.89	1.00
25	0.2	5.54	100%	0.59	0.23	0.93	0.85	0.64	0.80	0.88	0.83
25	0.25	5.48	100%	0.52	0.22	1.70	1.06	1.12	0.98	0.93	1.05
50	0.05	9.44	100%	0.91	0.07	0.09	0.35	0.05	0.30	2.00	1.55
50	0.1	10.02	100%	0.65	0.23	1.19	2.14	0.21	0.45	2.00	1.54
50	0.15	9.87	100%	0.70	0.26	1.26	1.02	1.02	0.93	1.92	1.42
50	0.2	9.54	100%	0.62	0.26	1.84	1.32	1.52	1.25	1.88	1.49
50	0.25	9.21	100%	0.53	0.30	2.72	1.47	2.81	1.58	2.62	1.93
100	0.05	17.38	100%	0.89	0.10	0.04	0.19	0.00	0.00	3.36	2.00
100	0.1	17.83	100%	0.58	0.24	3.86	5.06	0.91	0.93	3.02	1.79
100	0.15	17.15	100%	0.62	0.24	2.29	1.41	1.92	1.19	3.31	2.19
100	0.2	12.30	78%	0.45	0.32	2.70	2.07	3.77	1.81	4.00	2.14
100	0.25	15.82	59%	0.29	0.35	1.86	2.20	3.63	1.74	4.24	2.19
150	0.05	25.15	100%	0.88	0.08	0.08	0.27	0.02	0.14	4.81	2.79
150	0.1	24.83	100%	0.62	0.24	5.28	4.38	1.25	1.00	4.53	2.67
150	0.15	20.28	85%	0.47	0.29	2.81	1.83	3.34	1.71	4.81	2.53
150	0.2	20.29	76%	0.36	0.37	1.95	2.09	3.22	1.92	4.76	2.03
150	0.25	22.45	52%	0.15	0.33	0.68	1.51	4.28	1.18	5.45	1.26

### 5.4. Application to a Real Mine

To test the node distance metric in real mines for navigation purposes, we applied it to the topology graph of the Kaatiala mine (see Figure 3). The map was created manually using information from human divers. First, each map node was compared with all other nodes. The results are shown in Table 5. It is interesting to compare nodes with the same number of exits. For instance, nodes 2 and 5 have a similarity of 0.195, due to the differences in the features of their exits (height, width, horizontal direction, vertical direction). Similarly, nodes 4, 8, 9, and 10 show bad similarity even if they have the same number of exits. Note how nodes 15 and 16 have a very high similarity index. The distance formula returns a default value of 0.2 for the similarity index $E_{s,m}$ for all pairs of nodes s,m with a different number of exits.

Next, we repeated the calculation adding 25% of noise to the sensor measurements. Table 6 shows the results. Regardless of the noise, the best similarity index still refers to the correct node, although cases start to appear (for instance, between nodes 15 and 16) wherein the index is very similar and could therefore lead to localization errors.

Finally, to test the navigation, we conducted tests from the mine entrance to the outermost nodes (14, 15, 16), with different levels of noise. Table 7 summarizes the results. In all tests, the robot was able to successfully navigate from the entrance to the destination nodes, and only in a small number of cases, induced by the noise level added to the sensors, it had to perform a rollback operation to find the correct path. Clearly, the confidence level decreases with increasing noise. However, due to the small size of the graph, the robot did not perform any reckon procedure in most cases.

## 6. Conclusions

In this paper, we have presented a node matching procedure, a self-localization procedure, and a reckon procedure on labeled graphs under uncertainty, applied to autonomous robot topological navigation.

From the results of the numerical simulations conducted to evaluate the behavior and robustness of the proposed strategy, we can conclude that the two research questions have been satisfactorily addressed.

The proposed node-matching metric is effective for navigating under severe uncertainty, allowing the robot to recognize the correct node with a success rate of around 90% or higher with noise up to 25%, significantly decreasing for extremely high uncertainty. This increases to 100% with the application of the proposed *rollback* and *recknon* procedures during navigation, demonstrating how such procedures effectively aid autonomous navigation. Finally, as expected, the detection of particular visual features, the objects of interest, effectively helps the robot to correctly find its position in the topology graph, increasing the success rate and decreasing the need for the reckon steps.

The application of our procedure to the topological map of a real mine confirmed the effectiveness of the proposed approach. Although relatively small, this map displays the features of real mines, where the network of tunnels follows the ore and, therefore, shows certain irregularities that make localization easier due to the geometrical differences of the nodes.

The long-term goal of our research is to integrate the proposed strategies into the UX-1 Guidance and Navigation software. This will significantly improve its autonomous navigation capabilities, a key feature for future missions. The steps toward this goal include integrating additional information, such as tunnel and OOI data, to improve the algorithm’s robustness and its ability to understand its surroundings with increased confidence. In addition, the topological navigation module will need to be integrated with the low-level SLAM modules and tested first in hardware-in-the-loop experiments using data from previous missions and then in controlled real-world environments. This will allow for the testing of the complete navigation system and ensure its effectiveness in various scenarios.

In conclusion, our proposed approach is a promising solution for autonomous robot navigation in topological environments with uncertainty. In almost all cases, except for extremely high noise levels that led to high uncertainties and large graphs, the robot was able to successfully navigate from the starting node to the end node.

## Figures and Tables

**Table 5:** Value of similarity index $E_{s,m}$ for all the pairs of nodes of Kaatiala mine map (see Figure 3).

s/m	1	2	3	4	5	6	7	8	9	10	11	12	13	14	15	16
1	1	0.2	0.2	0.2	0.2	0.2	0.2	0.2	0.2	0.2	0.40	0.02	0.0	0.0	0.0	0.0
2		1	0.2	0.2	0.19	0.2	0.2	0.2	0.2	0.2	0.2	0.2	0.2	0.2	0.2	0.2
3			1	0.2	0.2	0.2	0.474	0.2	0.2	0.2	0.2	0.2	0.2	0.2	0.2	0.2
4				1	0.2	0.234	0.2	0.34	0.28	0.02	0.2	0.2	0.2	0.2	0.2	0.2
5					1	0.2	0.2	0.2	0.2	0.2	0.2	0.2	0.2	0.2	0.2	0.2
6						1	0.2	0.01	0.0	0.01	0.2	0.2	0.2	0.2	0.2	0.2
7							1	0.2	0.2	0.2	0.2	0.2	0.2	0.2	0.2	0.2
8								1	0.01	0.02	0.2	0.2	0.2	0.2	0.2	0.2
9									1	0.24	0.2	0.2	0.2	0.2	0.2	0.2
10										1	0.2	0.2	0.2	0.2	0.2	0.2
11											1	0.02	0.0	0.01	0.0	0.0
12												1	0.0	0.10	0.0	0.0
13													1	0.0	0.36	0.32
14														1	0.0	0.0
15															1	0.89
16																1

**Table 6:** Value of similarity index $E_{s,m}$ for all the pairs of nodes of Kaatiala mine map, with noise level of 25%.

s/m	1	2	3	4	5	6	7	8	9	10	11	12	13	14	15	16
1	0.56	0.2	0.2	0.2	0.2	0.2	0.2	0.2	0.2	0.2	0.34	0.01	0	0.0	0.0	0.0
2		0.71	0.2	0.2	0.15	0.2	0.2	0.2	0.2	0.2	0.2	0.2	0.2	0.2	0.2	0.2
3			0.69	0.2	0.2	0.2	0.23	0.2	0.2	0.2	0.2	0.2	0.2	0.2	0.2	0.2
4				0.69	0.2	0.11	0.2	0.25	0.25	0.01	0.2	0.2	0.2	0.2	0.2	0.2
5					0.87	0.2	0.2	0.2	0.2	0.2	0.2	0.2	0.2	0.2	0.2	0.2
6						0.75	0.2	0.01	0	0.01	0.2	0.2	0.2	0.2	0.2	0.2
7							0.79	0.2	0.2	0.2	0.2	0.2	0.2	0.2	0.2	0.2
8								0.75	0.01	0.01	0.2	0.2	0.2	0.2	0.2	0.2
9									0.81	0.23	0.2	0.2	0.2	0.2	0.2	0.2
10										0.78	0.2	0.2	0.2	0.2	0.2	0.2
11											0.45	0.02	0.0	0.01	0.0	0.0
12												0.52	0.0	0.11	0.0	0.0
13													0.62	0.0	0.34	0.27
14														0.60	0.0	0.0
15															0.85	0.74
16																0.70

**Table 7:** Navigation tests for the Kaatiala Mine: Success rate (SR), final value of the confidence index, number rollback, and reckon operations.

Destination Node												
14				15				16				
Noise		Final	Roll-			Final	Roll-		SR	Final	Roll-	
Level	SR	Conf.	Back	Reckon	SR	Conf.	Back	Reckon	SR	Conf.	Back	Reckon
0%	100%	1	0	0	100%	1	0	0	100%	1	0	0
10%	100%	0.75	0	0	100%	0.73	0	0	100%	0.84	1	0
25%	100%	0.54	0	0	100%	0.56	0	0	100%	0.43	1	1

## Data Availability

Data sharing is not applicable to this article (the data used have been randomly generated).

## Abbreviations

The following abbreviations are used in this manuscript:

AUV	Autonomous Underwater Vehicle
UUV	Unmanned Underwater Vehicle
UNEXMIN	UNderwater EXplorer for flooded MINes
UNEXUP	UNEXMIN Upscaling
ROV	Remotely Operated Vehicle
GNC	Guidance, Navigation, and Control
SLAM	Simultaneous Localization and Mapping
SF	Sensor Fusion
LLC	Low-Level Control
SIL	Software-In-the-Loop
HIL	Hardware-In-the-Loop
DVL	Doppler Velocity Log
IMU	Inertial Measurement Unit
SLS	Structured Light System
ROS	Robot Operating System
